# Glomerular Hypertrophy and Splenic Red Pulp Degeneration Concurrent with Oxidative Stress in 3xTg-AD Mice Model for Alzheimer’s Disease and Its Exacerbation with Sex and Social Isolation

**DOI:** 10.3390/ijms25116112

**Published:** 2024-06-01

**Authors:** Juan Fraile-Ramos, Josep Reig-Vilallonga, Lydia Giménez-Llort

**Affiliations:** 1 Institut de Neurociències, Universitat Autònoma de Barcelona, 08193 Barcelona, Spain; juan.fraile@uab.cat; 2 Department of Psychiatry and Forensic Medicine, School of Medicine, Universitat Autònoma de Barcelona, 08193 Barcelona, Spain; 3 Department of Anatomy, School of Medicine, Universitat Autònoma de Barcelona, 08193 Barcelona, Spain; josep.reig@uab.cat

**Keywords:** Alzheimer’s disease, peripheric organs, kidney, spleen, amyloidosis, oxidative stress

## Abstract

The continuously expanding field of Alzheimer’s disease (AD) research is now beginning to defocus the brain to take a more systemic approach to the disease, as alterations in the peripheral organs could be related to disease progression. One emerging hypothesis is organ involvement in the process of Aβ clearance. In the present work, we aimed to examine the status and involvement of the kidney as a key organ for waste elimination and the spleen, which is in charge of filtering the blood and producing lymphocytes, and their influence on AD. The results showed morphological and structural changes due to acute amyloidosis in the kidney (glomeruli area) and spleen (red pulp area and red/white pulp ratio) together with reduced antioxidant defense activity (GPx) in 16-month-old male and female 3xTg-AD mice when compared to their age- and sex-matched non-transgenic (NTg) counterparts. All these alterations correlated with the anxious-like behavioral phenotype of this mouse model. In addition, forced isolation, a cause of psychological stress, had a negative effect by intensifying genotype differences and causing differences to appear in NTg animals. This study further supports the relevance of a more integrative view of the complex interplay between systems in aging, especially at advanced stages of Alzheimer’s disease.

## 1. Introduction

The field of Alzheimer’s disease (AD) has continuously expanded since its inception. Since the first neurophysiological findings, specifically the accumulation of the amyloid-beta (Aβ) protein in plaques and tau neurofilaments, exhaustive efforts have been made to characterize the changes in the brain and their causes. New approaches have also considered additional alterations involved in this disease, such as oxidative stress, reactive glia, and changes in microglia, as important factors involved in the generation and progression of this disease [1]. However, one of the lines of research yet to be expanded in this field is the communication between the brain and the rest of the body during the progression of the disease and its effects. Thus, new investigations challenge the classical view with a more systemic approach to AD. The involvement of the peripheral immune system in neuroinflammation, which is a central feature of AD, has already been discussed, and crosstalk has been established [2,3,4]. In addition, peripheral tissues and organs have the capacity to clear Aβ derived from the brain [5]. This indicates that the peripheral organs are involved in the development and progression of AD, suggesting a new possible valid therapeutic approach for AD by enhancing peripheral Aβ clearance.

The main connections established so far pose the neuro-immunoendocrine and other homeostatic systems as major AD-vulnerable peripheral systems. Previous studies by our group have already determined an altered status of the liver at advanced stages of the disease. Amyloidosis in the hepatic blood vessels and the presence of oxidative stress and inflammation support the hypothesis of the involvement of the liver–brain axis in this neurodegenerative disease [6,7,8]. In addition to the liver, which is capable of degrading Aβ and excreting it in bile [9], other organs such as the kidney and the spleen have been seen to have amyloid depositions in systemic amyloidosis and may be affected during disease progression [10]. Renal function has been linked to the risk of dementia on many occasions. Patients with chronic kidney disease (CKD) are likely to present cognitive disorders and dementia [11,12]. Moreover, the central role of the kidney in the clearance of Aβ by excretion in the urine has strongly supported the use of dialysis as a possible therapeutic intervention [13]. A reduction in Aβ plasma levels in humans and the attenuation of AD phenotypes in the APP/PS1 model has been demonstrated after peritoneal dialysis [14]. On the other hand, the spleen has also been related to AD pathology. The spleen can affect the brain’s function through immune modulation, supporting a communication pathway between these two organs [15]. Moreover, alterations in inflammatory cytokines associated with splenic dysfunction in the APP/PS1/Tau model of AD also support the involvement of the spleen in AD [16].

Chronic stress has been classically described as the hyperactivation of the hypothalamic–pituitary–adrenal (HPA) axis and cellular inflammatory oxidative stress responses [17,18]. But chronic stress is also able to accelerate the neurodegeneration process of AD, affecting some of its features, such as the accumulation of β-amyloid, oxidative stress, inflammation, and the impairment of mitochondrial function [19]. In this context, social isolation, as an extrinsic environmental factor associated with psychological stress, can affect cognitive status and the development and prognosis of dementia. This fact has gained attention since the COVID-19 pandemic when imposed social isolation exerted a negative impact [20]. We recently demonstrated that male 3xTg-AD mice were strongly affected by naturalistic isolation (derived from the death of home cage mates), as shown by disrupted behavior and the liver, spleen, and kidney organometrics [21]. Thus, further study of behavioral and peripheral interplay under socially stressful scenarios can bring a better understanding of intrinsic and extrinsic vulnerability in aging and disease.

The 3xTg-AD mouse model has been extensively used for AD research; this model offers both neuropathological hallmarks of the disease, Aβ plaques, and tau tangles in an age-related manner [22]. Moreover, these mice also exhibit the prominent neuropsychiatric-like profile of the disease, which can already be recorded from the age of 4–6 months [23,24,25]. Taking advantage of these characteristics, our precedent work demonstrates hepatic oxi-inflammation and neophobia as potential targets for AD, with a strong sensitivity to sex and isolation [6]. The present work aims to further study the contribution of other peripheral–brain axes, namely the kidney and spleen axis, at advanced stages of the disease and how social isolation and stressful scenarios can impact this.

## 2. Results

### 2.1. Physical Metrics of Kidney and Spleen

**Figure 1:**
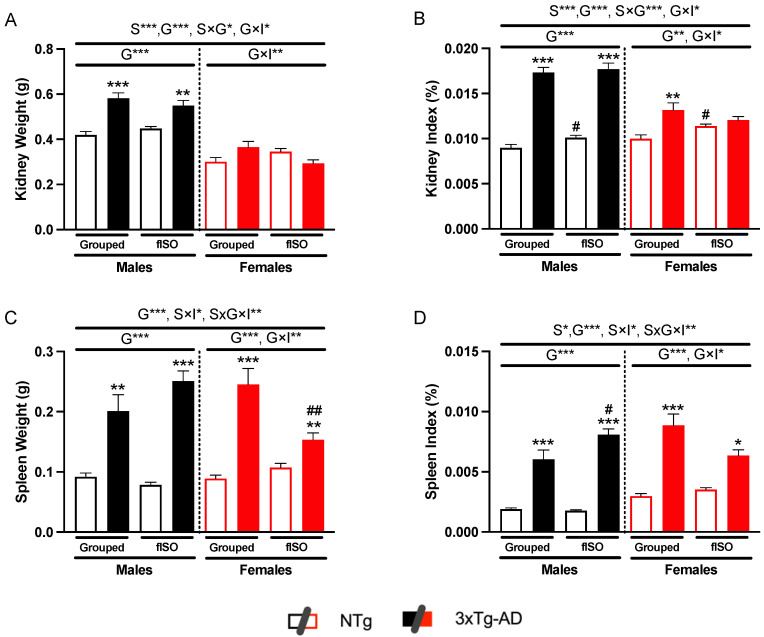
Physical status: kidney weights (**A**), kidney index (**B**), spleen weight (**C**), and spleen index (**D**). n = 5–10 mice per group, mean ± SEM. Sex: S*, *p* < 0.05; S***, *p* < 0.001. Genotype: G**, *p* < 0.01; G***, *p* < 0.001. Forced isolation: I***, *p* < 0.001. Interaction between sex and genotype: S × G*, *p* < 0.05; S × G***, *p* < 0.001. Interaction between genotype and isolation: G × I*, *p* < 0.05; G × I***, *p* < 0.001. Interaction between sex and isolation: S × I*, *p* < 0.05; S × I**, *p* < 0.01. Interaction between sex, genotype and isolation: S × G × I*, *p* < 0.05; S × G × I**, *p* < 0.01. Differences between two counterparts for genotype: * *p* < 0.05, ** *p* < 0.01, *** *p* < 0.001 vs. NTg counterpart; isolation: # *p* < 0.05, ## *p* < 0.01 vs. grouped counterpart.

The first measurement was the weight of both organs. In the case of the kidney, sex, and genotype effects were found both in the weight of the organ and the index (Figure 1A,B, S, all F’s (1,56) > 22.61, *p* < 0.0001; G, all F’s (1,56) > 15.79, *p* = 0.0002). In addition, the genotype had an interaction effect with sex (Figure 1A,B, all F’s (1,56) > 23.61, *p* < 0.0001) and isolation (Figure 1A,B, all F’s (1,56) > 4.062, *p* = 0.0487). Males had larger kidneys than females, and 3xTg-AD males had larger kidneys than NTg males. These differences were more marked in the index, where 3xTg-AD males showed a larger index, and females showed slight significance. Moreover, an isolation-dependent increase was observed in the kidney index of the NTg animals.

As for the spleen, the most marked effect was observed for the genotype (Figure 1C,D, all F’s (1,53) > 78.48, *p* = 0.0004). 3xTg-AD mice had twice the weight compared to NTg mice. Interaction effects between all factors and between sex and isolation (Figure 1C, S × I F (1,53) = 8.827, *p* = 0.0062; S × G × I F (1,53) = 10.02, *p* = 0.0026) were also present because of the reduction in the spleen’s weight in the 3xTg-AD isolated females. This effect was also present in the spleen index, although it did not reach statistical significance. The index showed a sex effect (Figure 1D, F (1,53) = 4.978, *p* = 0.0299) and an interaction effect (Figure 1D, S × I F (1,53) = 4.852, *p* = 0.032; S × G × I F (1,53) = 8.835, *p* = 0.0044). All 3xTg-AD animals had a higher spleen index than NTg animals, and isolated 3xTg-AD males had a higher index than non-isolated males. These results indicate that 3xTg-AD animals have renomegaly and splenomegaly.

### 2.2. Histopathological Assessment: H&E and Congo Red Staining

**Figure 2:**
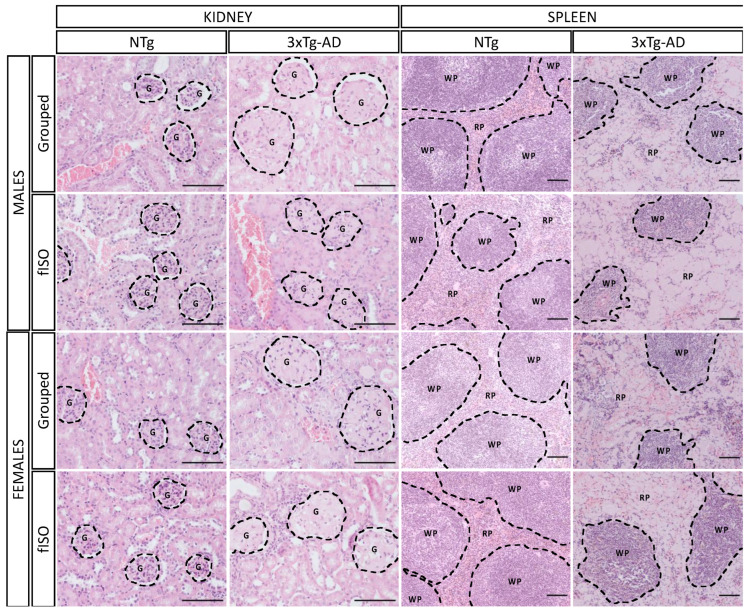
Histopathological assessment. Morphological comparison of kidney and spleen tissue in NTg and 3xTg-AD males and females for mice grouped and those submitted to forced isolation. H&E stained. Dashed lines indicate altered morphological features as follows: G, glomeruli; WP, white pulp; and RP, red pulp. The images were taken with ×20 and ×10 objective lenses; magnification bar = 100 μm.

**Figure 3:**
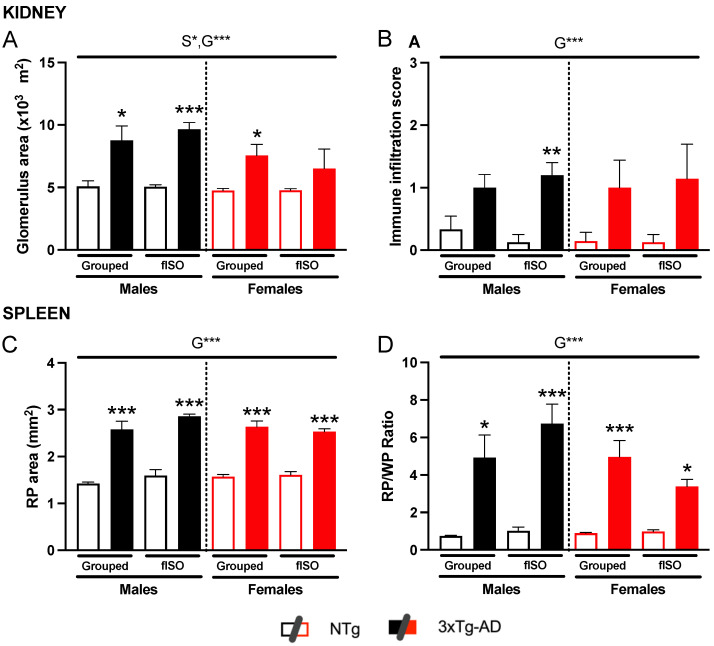
Quantification of different histopathological aspects. (**A**) Quantification of the glomerulus area and at least 20 glomeruli from each mouse were quantified. (**B**) Immune infiltration score of the kidney comprising the grades 0, none; 1, mild; 2, moderate; and 3, severe. (**C**) Quantification of the red pulp area and at least three different areas from each spleen were quantified. (**D**) The ratio between the red pulp area and white pulp area; at least three different areas from each spleen were quantified. n = 6–10 mice per group, mean ± SEM. Sex: S*, *p* < 0.05. Genotype: G***, *p* < 0.001. Differences between two counterparts for genotype: * *p* < 0.05,** *p* < 0.01, *** *p* < 0.001 vs. NTg counterpart.

The kidney and spleen sections were prepared and stained for microscopic analysis to determine morphological and physiological alterations (Figure 2). The kidney showed a marked alteration of the glomeruli in the 3xTg-AD animals. In NTg animals, the glomeruli were round and small, but in 3xTg-AD, their size was considerably higher, and their round form was lost. Quantitative analysis of this alteration measuring the glomerular area showed a sex and genotype effect (Figure 3A; S, F (1,57) = 4.31, *p* = 0.042; G, F (1,57) = 28.9, *p* < 0.0001). 3xTg-AD animals had higher glomerular areas than NTg animals, and this effect was more marked in males than females. Another aspect taken into account was the immune infiltration. which had genotype differences (Figure 3B, F (1,57) = 19.16, *p* < 0.0001) that were significantly higher in 3xTg-AD animals. On the other hand, the spleen also presented structural changes in the red pulp; the typical microarchitecture of the spleen was lost when the red pulp enlarged and disintegrated, making it look clear and almost disappear. As for the spleen, the quantification of the red pulp area showed a clear genotype effect (Figure 3C; F (1,53) = 240.1, *p* < 0.001), with all 3xTg-AD animals showing wider red pulp areas. The ratio of the red pulp and white pulp in the spleen also showed this enlargement of the red pulp according to genotype (Figure 3D; F (1,53) = 59.12, *p* < 0.001); in the ratio, a tendency was also observed in isolated males to have higher RP/WP indexes, whereas isolated females showed a lower RP/WP index.

**Figure 4:**
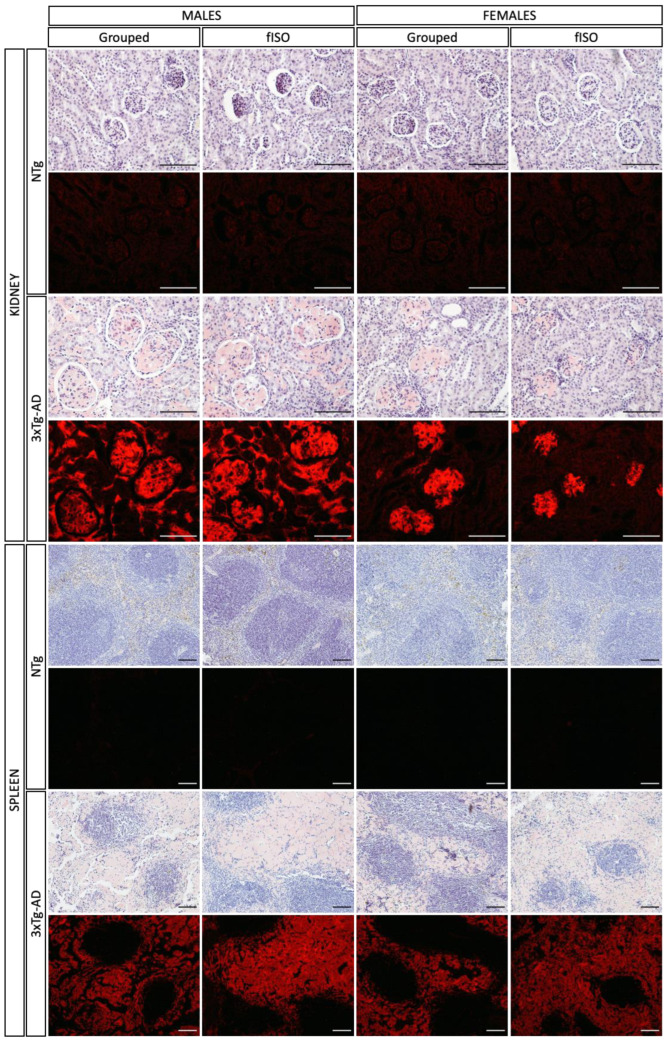
Amyloidosis assessment. Differences in amyloidosis in kidney and spleen tissue in NTg and 3xTg-AD males and females both grouped and those submitted to forced isolation. Congo red-stained. The images were taken with a ×20 and x10 objective lens; magnification bar = 100 μm.

Deepening the histopathological analysis of the kidney and spleen, staining with Congo red was performed to determine amyloidosis (Figure 4). In the case of the kidney, the staining marked the kidneys of 3xTg-AD animals exclusively with that characteristic reddish color. In addition, fluorescence images showed a more highly stained area in males than in females; in any case, the affected area was always the glomeruli. In the spleen, the result was similar; only 3xTg-AD animals presented Congo red-stained areas, and in accordance with the H&E staining, the area affected was the red pulp. It can be seen in the bright field image that the red pulp has a reddish tone, and focusing on the fluorescent images, the red pulp clearly shows red fluorescence. Thus, 3xTg-AD animals presented amyloidosis in the kidney (glomeruli) and the spleen (red pulp).

### 2.3. Oxidative Stress Status

The oxidative status of the kidney and spleen was determined based on the GSH cycle, considering the levels of GSH and the activity of the antioxidant enzymes involved in the process (GPx and GR).

**Figure 5:**
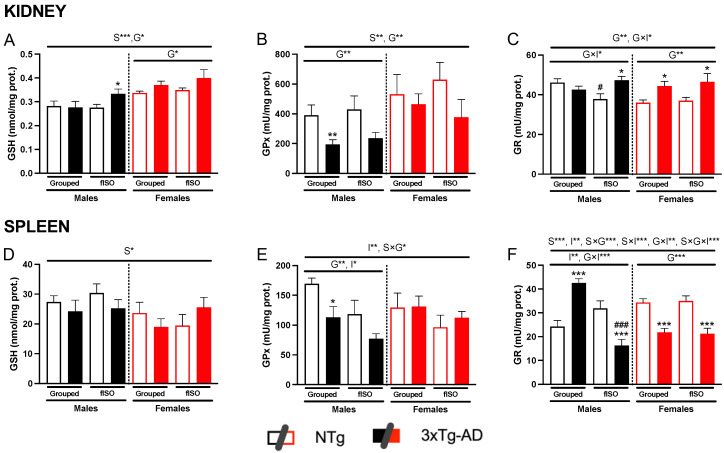
Oxidative stress in peripheral organs: kidney GSH levels (**A**), GPx activity (**B**), GR activity (**C**), spleen GSH levels (**D**), GPx activity (**E**), and GR activity (**F**). n = 5–10 mice per group, mean ± SEM. Sex: S*, *p* < 0.05; S**, *p* < 0.01; S***, *p* < 0.001. Genotype: G*, *p* < 0.05; G**, *p* < 0.01; G***, *p* < 0.001. Forced isolation: I*, *p* < 0.05; I**, *p* < 0.01. Interaction between sex and genotype: S × G*, *p* < 0.05; S × G***, *p* < 0.001. Interaction between genotype and isolation: G × I*, *p* < 0.05; G × I**, *p* < 0.01; G × I***, *p* < 0.001. Interaction between sex and isolation: S × I***, *p* < 0.001; S × I**, *p* < 0.01. Interaction between sex, genotype, and isolation: S × G × I***, *p* < 0.001. Differences between two counterparts for genotype: * *p* < 0.05, ** *p* < 0.01, *** *p* < 0.001 vs. NTg counterpart; isolation: # *p* < 0.05, ### *p* < 0.001 vs. grouped counterpart.

Focusing first on the kidney, GSH levels presented sex and genotype differences (Figure 5A; S, F (1,57) = 22.86, *p* < 0.0001; G, F (1,57) = 5.105, *p* < 0.028), with females having higher levels than males, and 3xTg-AD animals having higher levels than NTg. The activity of the antioxidant enzymes also showed changes according to genotype (Figure 5B,C, all F’s (1,51) > 11.45, *p* = 0.0014). These differences consisted of a reduction in GPx activity and an increase in GR activity in the 3xTg-AD animals. In the case of GPx, sex differences were also present (Figure 5B, F (1,55) = 9.56, *p* = 0.003), and the enzyme activity was higher in females, while only males achieved statistical significance by a reduction in this enzyme’s activity. In the kidney, the only effect of the isolation was the interaction effect with the genotype (Figure 5C, F (1,51) = 4.11, *p* = 0.048) shown by the Ntg isolated males.

As for the spleen, GSH levels only showed sex differences (Figure 5D, F (1,50) = 4.47, *p* = 0.039); in this case, males tended to have higher levels than females. GPx activity presented isolation differences (Figure 5E, F (1,53) = 7.338, *p* = 0.009), with all isolated animals showing a slight reduction in GPx activity. In addition, males also presented genotype differences (Figure 5E, F (1,27) = 8.06, *p* = 0.0085), as 3xTg-AD males presented lower activity levels than NTg males. GR activity showed a clear genotype effect, which differed according to sex (Figure 5F; S × G, F (1,55) = 18.56, *p* < 0.001). In males, GR activity acted differently depending on isolation (Figure 5F; G × I, F (1,30) = 41.61, *p* < 0.001); in grouped males, 3xTg-AD had higher levels of activity than NTg, whereas, in isolation, 3xTg-AD males had lower levels of activity than NTg males. In females, genotype differences were the same regardless of the social conditions (Figure 5F; G, F (1,25) = 43.91, *p* < 0.001). 3xTg-AD females all presented a reduction in the activity of GR when compared to NTg females.

### 2.4. Statistical Correlation between the Studied Variables

**Figure 6:**
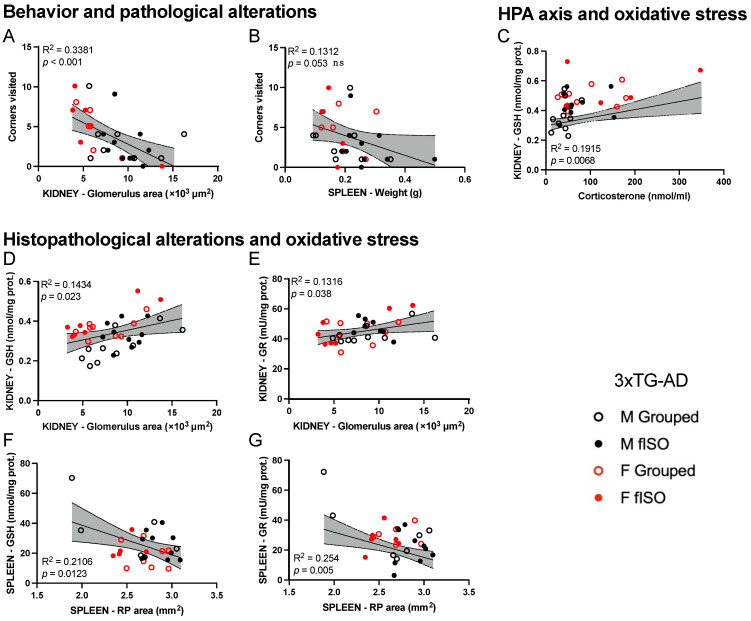
Correlation between the studied variables of the kidney and the spleen in 3xTg-AD animals. Correlations from behavior and histopathological alterations corresponding to the number of visited corners and glomerulus area (**A**) in the kidney and spleen weight (**B**) In the spleen, correlations between the HPA axis and oxidative stress corresponding to the levels of GSH in the kidney and serum levels of corticosterone (**C**). Correlations between the histopathological alterations and the oxidative stress corresponding to the glomerulus area in the kidney, the levels of GSH (**D**) and GR activity (**E**) and the red pulp (RP) area in the spleen, and levels of GSH (**F**) and GR activity (**G**).

The statistical analysis of the correlations of the variables was also included in this study for each organ, with the behavioral and corticosterone data measured for the same mice in a previous study [6]. In Figure 6A–G, we can see the correlations from 3xTg-AD animals established between behavioral variables, corticosterone levels, metrics variables, histopathological variables, and oxidative stress variables. These correlations were not present in NTg animals.

Neophobia behavior shown by the number of visited corners correlated with histopathological (glomerulus area) in the kidney (Figure 6A), indicating a lower exploration rate as the glomerular area is larger. In the spleen, the correlation between behavioral variables and physical disturbances did not reach statistical significance, but they did show the same tendency (Figure 6B). The HPA axis showed a relationship with the oxidative stress status as the levels of corticosterone were related to the levels of GSH in the kidney (Figure 6C). Finally, all these histopathological alterations also correlated with oxidative stress status. In the case of the kidney, the enlargement of the glomeruli positively correlated with GSH levels (Figure 6D) and GR activity (Figure 6E), indicating higher oxidative stress defense with a higher glomerulus area. In the case of the spleen, however, the red pulp enlargement negatively correlated with GSH levels (Figure 6F) and GR activity (Figure 6G), indicating a reduction in antioxidant defenses with higher RP areas.

## 3. Discussion

AD is gaining relevance within an aging world as the most important neurodegenerative disease and form of dementia. This disease has a significant impact on the organism beyond cognitive impairment. However, most of the research has focused on changes in the brain, whereas the involvement of peripheral organs has been scarcely investigated until now. Recent studies suggest that Alzheimer’s disease also has a systemic component, producing metabolic dysfunction and different pathological processes in peripheral organs [26]. We have recently provided evidence that the liver is affected in 3xTg-AD mice, and we propose hepatic amyloidosis and the dysregulation of its oxi-inflammatory defenses as potential targets for AD [6]. In this systemic approach to AD, here, we further investigate the histopathological profile and the oxidative stress status of the kidney and the spleen of these animals. Current findings show how morphological, structural, and oxidative stress alterations in these two organs further support the relevance of a peripheral organ–brain axis in the integrative system understanding of AD. In addition to the genotype differences, compared to the same age gold standard of C57BL/6 mice with physiological aging, the impact of sex and forced isolation as intrinsic (biological) and extrinsic (social conditions) factors, respectively, were also demonstrated.

### 3.1. Physical and Histopathological Alterations of Kidney and Spleen

Abnormalities were found in physical measurements and the histopathological analysis of the kidney and spleen of 3xTg-AD mice compared to NTg mice. Their weight and index (relative weight) were considerably high, denoting the hypertrophy of both organs, mostly in the spleen, in both sexes of 3xTg-AD mice. The kidney, with an important function in maintaining the homeostasis of the organism as it is currently, is also considered to have a role in the peripheral clearance of β-amyloid [13,27]. In the case of the spleen, the splenomegaly found can be important since it has been usually related to serious systemic diseases [28,29]. In previous works in mouse models for AD, splenomegaly has become a common feature and is related to inflammation and autoimmune processes [16,30]. Considering that the spleen also acts as a blood filter and is attributed to the physiological cleansing of Aβ [31], this splenomegaly supports the idea that the organs related to this function are the most affected in AD-pathogenic conditions.

In the present work, histopathological analysis showed physical abnormalities in both organs. In the case of the kidney, the increase in the glomeruli is almost twice its normal size, and the loss of its typical round morphology were significant features associated with the AD phenotype. On the other hand, the spleen presented structural destruction regarding the red pulp. This was consistent with previous results from 19-month-old 3xTg-AD males [7], and it is now seen that these alterations occur in both sexes, with higher severity in males than in females at advanced stages of the disease.

Congo red staining revealed that these histopathological changes were due to the accumulation of Aβ in the glomeruli of the kidney and the red pulp of the spleen. Therefore, these observations mimic the morphological alterations observed in patients with systemic amyloidosis as Aβ infiltration was seen in the glomeruli from kidney biopsies [32]. Moreover, unilateral nephrectomy has been shown to increase the deposition of Aβ in the brain, aggravating AD pathologies in the brain [33]. In the case of the spleen, the accumulation of Aβ has been seen in the mouse models of AD [34], and its potential to physiologically clear Aβ from the blood suggests that splenectomy could aggravate AD pathogenesis [31]. However, this effect has not yet been constated in humans. Moreover, in other studies performed with the 3xTg-AD mouse model, severe splenomegaly and structural destruction were reported [16]. This could indicate systemic amyloidosis in 3xTg-AD animals and pose the kidney and the spleen as main targets for its clearance. In fact, it is known that β-amyloid efflux from the brain to the periphery via the Blood–brain barrier (BBB) occurs during the disease for peripheral clearance, contributing to the brain’s mechanisms for the removal of Aβ [5]. In advanced stages of the disease, the BBB of 3xTg-AD mice was disrupted, and its permeability increased, allowing even more Aβ to reach the blood flow and the periphery [35,36]. The results of the present study further demonstrate the disruption or saturation of these clearing systems, supporting the clearing of brain Aβ and leading to its accumulation in the peripheral organs at the age of 16 months.

### 3.2. Kidney and Spleen Oxidative Stress

GSH metabolism has been widely used as a biomarker of oxidative stress along with the activity of both GR and GPx activity. The imbalance in the levels of GSH and the activity of these enzymes is considered to be a sign of oxidative stress in the cell [37,38]. Therefore, molecularly deepening in both organs, the level of oxidative stress was determined, and the results showed that the previous observations in the liver were reproduced in the kidney and spleen [6]. GSH levels remained fairly constant, although, in the kidney, they experienced a slight increase with regard to sex, with females having higher levels and genotypes, with 3xTg-AD animals tending to have more GSH. High levels of intracellular GSH are considered to be an adaptative response to oxidative stress [39].

In all organs, a decrease in GPx activity was observed in 3xTg-AD animals in both sexes, coinciding with studies in humans and animals where the decrease in the activity of this antioxidant enzyme is related to oxidative stress in this disease [40]. This oxidative stress was sex-dependent as it affected males more significantly. GR activity, despite having the most pronounced genotype differences, had opposite effects depending on the organ. In the case of the kidney, activity increased, possibly to compensate for the reduction in GPx activity. However, in the spleen, the activity of GR was also reduced, so in this organ, both enzymes showed lower activity. It should be noted that in the case of males, GR activity showed differences by social isolation, these differences pointing to greater oxidative stress in both organs. To better understand these systems and their interplay, the correlations between the different variables of organometrics, histopathology, cellular oxidative stress, neophobia, and HPA axis stress (corticosterone) were analyzed.

### 3.3. Correlations between Behavior, Histopathology, HPA Axis and Peripheral Oxidative Stress

In both organs, several significant correlations were found for the 3xTg-AD genotype but were not present in NTg mice. Anatomical and histopathological alterations correlated with neophobia, which is a fearful response to novelty, typically described as part of the neuropsychiatric-like phenotype of the 3xTg-AD mouse model [41,42,43]. The correlation with neophobia shows that both behavioral and peripheral changes happen at the same time in the advanced stages of the disease and agrees with previous results from the liver [6]. Moreover, these alterations were also related to oxidative stress defense changes, as shown by GSH levels and GR activity. Further correlations between histopathological and oxidative stress suggest that affectation due to Aβ in the glomeruli contributed to this oxidative stress and renal health. This is important since oxidative stress can affect kidney function and lead to renal damage [44,45]. Additionally, impaired kidney function has been associated with cognitive decline in older adults [46]. In the case of the spleen, despite splenomegaly and the destruction of the spleen’s microarchitecture being detected in the 3xTg-AD model on numerous occasions [7,16,26,47], this is the first time that red pulp is identified as a splenic structural target in AD. Noteworthy, the red pulp area correlated with GSH levels and GR activity in the same way as the glomerular area from the kidney.

Psychological stress during the development of this disease [48] can also affect the oxidative stress status of peripheral organs [49,50]. It has been found that chronic kidney disease can disturb cortisol regulation, leading to subclinical hypercortisolism and reduced cortisol clearance [51]. High cortisol levels may worsen cognitive status and become relevant to the generation and progression of AD [52]. In the present work, 3xTg-AD mice, but not NTg mice, showed a correlation between corticosterone and GSH levels, suggesting that corticosterone levels may be related to their increased oxidative stress. In fact, high levels of corticosterone have already been shown to produce oxidative stress in rats [53], and prolonged isolation stress has already been shown to accelerate the onset of AD in the 5xFAD mouse model [54].

Overall, these correlations, at different levels of study, indicate the active interplay between these organs and the brain. Further research about the dynamics of this communication under the AD pathological condition can allow for a broader but integrative vision of this neurodegenerative disease.

### 3.4. Study Limitations

Different limitations to this basic translational research study were found. Starting with the scarce knowledge of the non-classical roles of the peripheral organs in the involvement of AD limits the conceptual framework and discussion and creates demands for primary screening. Further biomarkers of organ functioning should have been included to confirm the histological and oxidative stress alterations. It would have also been interesting to evaluate other distinctive behavioral hallmarks and their relationship with peripheral changes in addition to examining the neurological changes happening in the brain. Only one model was used in this study, but other genetic and/or sporadic models of AD should be assessed, as well as different stages of the disease. Social isolation was assessed as an extrinsic factor, but other factors may also be affecting the modulation of these changes and should also be considered.

### 3.5. Future Perspective of Peripheral Organs in AD

Apart from the classical brain, structural and functional alterations in the onset of the disease [55], a more integrated vision of AD has already allowed for consideration of other hallmarks, such as neuroinflammation [56,57] and impaired brain bioenergetics [58]. In addition, clinical and basic research is now providing evidence of multiple connections between neuroinflammation and systemic inflammation [59], opening a third-generation approach to the disease that considers peripheric implications in AD. For instance, we have previously demonstrated the interplay of the immune system and behavior in the 3xTg-AD model [4,60]. The present and the previous work [6,8,61] show, at different levels of study, the implication of the liver, the kidney, and the spleen in Aβ pathology in AD. A clearly augmented relative weight in these organs, the histopathological alterations localized in the hepatic blood vessels, the renal glomeruli, and the splenic red pulp, as well as the reduction in oxidative stress defenses, stood out as the main alterations. However, the involvement of peripheral organs in AD is still a novel and little-explored idea. Therefore, identifying and quantifying these pathological peripheral signatures of AD can lead to new translational findings that may unveil their significance in the clinical field. In fact, blood and urine levels of Aβ have been considered as potential biomarkers for AD [62,63,64]. Similarly to classical visualization methods in organometry and other peripheral organ parameters used in the diagnosis of different diseases [61], the present results suggest that the relative weight of the liver, the kidney, and the spleen could be of use in AD diagnosis and non-invasive tracking. Also, new technologies of imaging, such as magnetic resonance imaging (MRI), could be useful not only to see the structural and functional organization of the brain [65] but also the peripheral organs [66] and their possible hypertrophy. Further research endeavors are needed to clarify the complex bidirectional communication between these brain–peripheral axes, posing a new and promising area of research. Similarly, under integrative system analysis, whether other systems can also take part in the development and progression of AD and be affected as a consequence of disease progression is yet to be determined.

## 4. Materials and Methods

### 4.1. Animals

Homozygous triple-transgenic 3xTg-AD mice carrying human PS1/M146V, APPSwe, and tauP301L transgenes were produced at the University of California, Irvine [67]. Briefly, two independent transgenes encoding human APPSwe and tauP301L, both regulated by the mouse Thy1.2 transcription factor, were co-injected into single-cell embryos from PS1KI (PS1M146V knock-in) mice. A cohort of sixty-six 16-month-old male and female mice from the Spanish colonies of homozygous 3xTg-AD mice (n = 37) and from litters of a breeding program established at Universitat Autònoma de Barcelona after embryonic transfer to a C57BL/6 strain background [68], from now on referred to as “3xTg-AD”, and C57BL/6 wildtype mice (n = 29), from now on referred to as “NTg”, non-transgenic mice, were used. All animals were housed in groups of 3–4 individuals of the same sex and genotype until the experiment. They were maintained in cages (Makrolon, 35 × 15 × 15 cm3) under standard laboratory conditions (12 h light/dark, cycle starting at 8:00 a.m., food and water ad libitum, 22 ± 2 °C, 50–60% humidity). Biochemical and histopathological analyses were performed blind to the experiment in a counterbalanced manner.

All procedures were performed according to the Spanish legislation on the ‘Protection of Animals Used for Experimental and Other Scientific Purposes’ and the EU Council directive (2010/63/EU) on this matter. The protocol CEEAH 3588/DMAH 9452 was approved on the 8th of March 2019 by the Departament de Medi Ambient i Habitatge, Generalitat de Catalunya. The ARRIVE guidelines developed by the NC3Rs were followed with the objective of reducing the number of animals used in experimental procedures [69].

### 4.2. Experimental Design

To mirror the translational psychological stressful scenario that was lived in during the COVID-19 pandemic, mice from both genotypes and sexes were subjected to a forced isolation (fISO) paradigm; at 13 months of age, the animals were separated and individualized in cages and were kept for 3 months until they were euthanized. In all the cases, even though isolated animals were in their cages and could not physically interact with others, they were still connected through olfaction and audition. Nesting conditions were the same as for the grouped animals.

### 4.3. Physical Status

Animals were weighed at the time of sacrifice, and the organs were dissected, weighed, and stored at −80 °C until protein extraction for biochemical analysis. The index of the organ or relative weight was calculated according to the following equation: Organ index = Organ weight (g) / Body weight (g).

### 4.4. Behavioral Phenotype

The corner test was used to evaluate the immediate neophobia response of the animals in a new standard home cage with a clean bed of wood save. The animals were placed in the center of the cage and observed for 30 s. The following information was recorded: the number of visited corners, the latency of the first rearing, and the number of rearings. Behavioral assessment was performed from 9:00 a.m. to 1:00 p.m., counterbalanced and blind to the experimenter.

### 4.5. Histopathological Assessment

After euthanasia, one kidney and half of the spleen were dissected, fixed with 10% formalin for 24 h, and embedded in paraffin for further histopathological analysis. Slices of 5 μm and 10 μm thickness were deparaffinized and re-hydrated by several passes through ethanol at different concentrations.

#### 4.5.1. Morphological and Structural Analysis with H&E

Standard staining with hematoxylin and eosin (H&E) (Sigma-Aldrich, San Luis, MO, USA) was performed. Briefly, a 5 min incubation with Harris hematoxylin and a 2 min incubation with Eosin Y was performed, with a dedifferentiation phase in between. Finally, the slices were manually mounted with a DPX mounting medium after dehydration. The images were taken with a ×20 objective on a Nikon Eclipse 80i microscope, using a digital camera running on the control software ACT-1 (ver2.70). For histopathological measurements in the kidney, the glomerular area of at least 20 glomeruli of each mouse was measured. In the spleen, three random areas across the spleen of each mouse were selected, and the area of red pulp and white pulp were quantified. In both cases, measurements were performed using Fiji software version 2.14.0/1.54f for image processing.

#### 4.5.2. Congo Red Staining for Amyloidosis

Amyloidosis was assessed using a Congo red kit (Sigma-Aldrich, San Luis, MO, USA) for amyloid staining. The procedure was followed as the kit indicated, with incubation in Mayer’s hematoxylin and then in Congo red solution. Finally, the slices were manually mounted with the DPX mounting medium after dehydration. The images were taken with a ×20 objective on a Nikon Eclipse 80i microscope under normal light (bright field) and fluorescence light (fluorescent filter G-2A), using a digital camera running on the control software ACT-1 (ver2.70).

### 4.6. Oxidative Stress Analysis

The antioxidant capacity of the organs was evaluated from homogenates of the kidney and spleen tissue by the levels of total glutathione (GSH), the main non-enzymatic reducing agent of the organism, and the enzymatic activity of glutathione peroxidase (GPx) and reductase (GR). This benefited from the enzymatic recycling method previously described [70]. All reagents from Sigma-Aldrich, San Luis, MO, USA.

Total GSH was determined by measuring the absorbance at 412 nm, which was adapted to 96-well plates with slight modifications [71].

The enzymatic activity of glutathione reductase was assessed following the method described by Massey and Williams [72] with slight modifications. The oxidation of NADPH was monitored spectrophotometrically and performed at 340 nm for 300 s.

The enzymatic activity of glutathione peroxidase was measured using the modified previously described technique [73,74]. The reaction was followed spectrophotometrically by a decrease in the absorbance at 340 nm for 300 s.

The results were expressed as milliunits (mU) of enzymatic activity per milligram (mg) of the organ protein.

### 4.7. Functional Correlates

Correlations between the variables measured in this study and the behavioral phenotype and HPA axis variables from the same animals [6] were carried out using Pearson’s test. The data are shown as scattered plots with the total correlation coefficient and the significance, as well as separated coefficients by genotype (NTg and 3xTg-AD).

### 4.8. Statistics

Results are expressed as mean ± SEM. GraphPad Prism 8.0. Three-way and two-way analyses for multiple variables (ANOVA) followed by Tukey’s post hoc test for multiple comparisons were used. Student’s *t*-test was used to assess the significance between two independent groups. In all the tests, statistical significance was considered at *p* < 0.05.

## 5. Conclusions

Taking advantage of the 3xTg-AD genotype and considering sex as an intrinsic factor and isolation as an extrinsic factor, alterations in peripheral organs were studied in the translational advanced stage scenario of the disease. The present work provides evidence of renal and splenic organometric and histopathological alterations accompanied by oxidative stress in the 3xTg-AD mouse model in a sex- (worse in males) and social condition- (worse in forced isolation) dependent manner. Renal glomeruli and the red pulp of the spleen were determined as key morphological and structural alterations accumulating Aβ, confirming that both organs were compromised in AD. The concurrent enhancement of oxidative stress was evidenced by a decrease in the activity of the antioxidant enzyme GPx in both organs. Furthermore, these alterations correlated with the 3xTg-AD animals’ behavioral phenotype. This work further highlights the relevance of the peripheral organ–brain axis in aging, especially in AD. It brings the research closer to taking a more comprehensive view of integrative systems where intrinsic (sex) and extrinsic (social conditions) factors play a role.

## Data Availability

The data presented in this study are available on request to the corresponding author.
